# 

**Published:** 1870-09

**Authors:** 


☞ NOTICE.—Subscriptions received during September will be credited for a whole year, beginning January 1st, 1871, and the numbers from now until then sent free—equal to *Four Months Free.*
☞ BACK NUMBERS from January, 1870, will be supplied to NEW subscribers desiring the current volume complete.

